# Training in High-Throughput Sequencing: Common Guidelines to Enable Material Sharing, Dissemination, and Reusability

**DOI:** 10.1371/journal.pcbi.1004937

**Published:** 2016-06-16

**Authors:** Bastian Schiffthaler, Myrto Kostadima, NGS Trainer Consortium, Nicolas Delhomme, Gabriella Rustici

**Affiliations:** 1 Department of Plant Physiology, Umeå Plant Science Centre, Umeå University, Umeå, Sweden; 2 Department of Haematology, University of Cambridge, Cambridge, United Kingdom; 3 Department of Forest Genetics and Plant Physiology, Umeå Plant Science Centre, Swedish University of Agricultural Sciences, Umeå, Sweden; 4 School of Biological Sciences, Department of Genetics, University of Cambridge, Cambridge, United Kingdom; Ontario Institute for Cancer Research, CANADA

## Abstract

The advancement of high-throughput sequencing (HTS) technologies and the rapid development of numerous analysis algorithms and pipelines in this field has resulted in an unprecedentedly high demand for training scientists in HTS data analysis. Embarking on developing new training materials is challenging for many reasons. Trainers often do not have prior experience in preparing or delivering such materials and struggle to keep them up to date. A repository of curated HTS training materials would support trainers in materials preparation, reduce the duplication of effort by increasing the usage of existing materials, and allow for the sharing of teaching experience among the HTS trainers’ community. To achieve this, we have developed a strategy for materials’ curation and dissemination. Standards for describing training materials have been proposed and applied to the curation of existing materials. A Git repository has been set up for sharing annotated materials that can now be reused, modified, or incorporated into new courses. This repository uses Git; hence, it is decentralized and self-managed by the community and can be forked/built-upon by all users. The repository is accessible at http://bioinformatics.upsc.se/htmr.

## Author Summary

In recent years, the advancement of high-throughput sequencing (HTS) and the rapid development of numerous analysis algorithms and pipelines in this field have resulted in an unprecedentedly high demand for training scientists in HTS data analysis. Generating effective training materials is time-consuming, and a large body of training materials on HTS data analysis has already been generated but is rarely shared among trainers. In this paper we provide guidelines to trainers for describing training materials to increase their reusability. The best practices standards proposed here have been used to annotate a collection of HTS training materials, which is now available to the trainers’ community in Git and discoverable through the ELIXIR and GOBLET portals. Efforts are now underway to utilize the strategy presented in this paper to annotate a wider collection of training materials and define a generic approach for the curation and dissemination of materials that should be adopted by existing training portals and new emerging initiatives.

This is part of the *PLOS Computational Biology* Education collection.

## Introduction

The advent of high-throughput sequencing (HTS) has revolutionized biological and biomedical research [1], allowing researchers to generate an overwhelming amount of genome-wide data. Now that sequencing is accessible to most, the major bottleneck has shifted from HTS data generation to analysis and interpretation. These remain challenging tasks due to the complexity of the analytical pipelines, the extensive list of available tools, and the evolving nature of the field. To support the need of researchers to carry out their data analysis, institutions worldwide offer highly specialized training on HTS data analysis (Table 1). However, despite the increase in the number of training solutions, the demand still largely exceeds what is currently offered.

**Table 1:** Training providers—among the trainers consortium—offering hands on training courses on the analysis of HTS data.

Institution	Target audience	URL
**Bioinformatics Facility, Umeå Plant Science Center, Umeå University and SLU, Umeå, Sweden**	Undergraduate, PhD students, Postgraduate, Professional development	https://bioinformatics.upsc.se
**Bioplatforms Australia/CSIRO**	PhD students, Postgraduate, Professional development	http://www.bioplatforms.com/current-training-courses/
**BITS Facility, VIB, Belgium**	PhD students, Postgraduate, Professional development	https://www.bits.vib.be/training
**CSC—IT Center for Science, Finland**	PhD students, Postgraduate, Professional development	https://csc.fi/web/training and http://chipster.csc.fi/
**ELIXIR-EE**	PhD students, Postgraduate, Professional development	http://elixir.ut.ee/Main/Courses
**ELIXIR-ITA/IIB**	Undergraduate, PhD students, Postgraduate, Professional development	http://bioinformaticstraining.pythonanywhere.com/
**EMBL-EBI**	Postgraduate, Professional development	https://www.ebi.ac.uk/training
**HPCBio, University of Illinois, USA**	PhD students, Postgraduate, Professional development	http://hpcbio.illinois.edu/content/hpcbio-workshops
**Science for Life Laboratory (SciLifeLab), Sweden**	PhD students, Postgraduate, Professional development	http://www.scilifelab.se
**SIB Swiss Institute of Bioinformatics, Switzerland**	PhD students, Postgraduate, Professional development	http://edu.isb-sib.ch/
**University of Cambridge, Cambridge, UK**	Undergraduate, PhD students, Postgraduate, Professional development	http://bioinfotraining.bio.cam.ac.uk/

Typically, the training offered by most institutions consists of short (2–6 days), intense courses, often focusing on a particular HTS pipeline—e.g., RNA-Seq, ChIP-Seq, whole genome sequencing, or variant analysis. During training, instructors and course organizers aim to provide a well-balanced mixture of lectures, which cover the data generation steps and illustrate the theory behind the analysis, and practical sessions, in which trainees can practice running HTS pipelines on real datasets [2]. Post-course surveys have revealed that participants regard the practical sessions as the most valuable components of a training course, as they represent an opportunity to run complex pipelines under the supervision of highly skilled trainers and discuss the issues associated with the analysis of such datasets with the experts in this field [3].

Years of experience in delivering such courses has taught us that the best-suited trainers are scientists who deal with HTS data analysis on a daily basis. Trainers can therefore include researchers working on HTS projects, computer scientists developing relevant algorithms and software, as well as bioinformaticians providing data analysis support to research groups. Consequently, for most instructors training is not formally part of their job and is done in addition to an already heavy workload.

Generating effective training materials (e.g., lectures and practical exercises) and testing them to ensure the smooth and successful delivery of a training course are time-consuming activities that all trainers need to undertake prior to a training event. In the last few years, a large body of training material on HTS data analysis has been generated; however, the sharing of such materials among trainers rarely happens, leaving instructors around the world with the need to constantly reinvent the wheel. Therefore, there is a need to develop mechanisms and best practices to both increase the visibility of complete training materials and enable their reusability, ensuring a reduction of trainers’ workload and fostering interactions within the trainers community.

Several initiatives have been established in recent years to support bioinformatics training and create community resources, most notably ELIXIR [4], a research infrastructure for coordinating biological data across Europe, which collaborates with global efforts such as GOBLET [5,6] to ensure that an adequate provision of training is put in place to reach a large and diversifying user base. Both initiatives are developing training portals (ELIXIR’s TeSS [7] and the GOBLET training portal [8,9]) to allow for collation of training materials, increasing their discoverability.

The establishment of such portals is of great importance, but this does not guarantee materials’ reusability. The development and production of training materials is usually undertaken by individual trainers for their personal use, often with a particular course or learning context in mind. As there is a sole developer and initial user of the materials, they are often lacking in detailed description or documentation, thereby making it difficult for another trainer to determine what the purpose of the training session was, who the materials were aimed at, and what resources are required to run such a session. Additionally, there is often great variety in the style of training materials presented (due to personal preference) and the level of detail in the content (e.g., PowerPoint slides annotated with notes).

If materials could be described consistently, so that either trainers or trainees understood what they could gain from using these materials, reusability of materials may be easier to achieve. For consistent description to take place, however, some form of guidance or best practice standard is required that allows for the addition of metadata to describe the materials and their use. HTS-related material also has additional issues when sharing is considered: (i) the constant evolution of the technologies that requires frequent materials revision; (ii) the incessant development of analysis tools, which prevents the establishment of standardized analytical procedures and training materials; and (iii) their complexity, as training materials are often linked to large datasets that require dedicated storage.

The ability to easily share materials via online portals is a fairly new phenomenon, and while providing guidance or best practice standards to trainers for describing their materials is a step in the right direction for more reusability, these also need to be adopted by those who provide the portals enabling the appropriate and correct information to be displayed.

This raises the following questions: What can trainers do to improve the sharing and reusability of their materials? What best practice can they adopt to enable the effective delivery of materials developed by another trainer?

To tackle these issues, on 13–14 January 2015, a workshop on “Best practices in next-generation sequencing data analysis” took place at the University of Cambridge, bringing together 29 trainers in the field of HTS data analysis, representing seven ELIXIR nodes and ten GOBLET partners (see S1 Table), with the aim to: (i) meet and discuss issues associated with the reusability of training materials, (ii) define a collective strategy to tackle such issues and identify an approach to the curation of training materials to enable their reusability among trainers, and (iii) implement this curation strategy by creating a unified collection of consistently described and well-annotated training materials.

This article provides a summary of the issues that were discussed during the workshop and presents the workshop’s major outcomes: the best practices guidelines that have been adopted for the curation of HTS training materials and a curated set/repository of materials that is now accessible to the entire training community through Git and is discoverable via TeSS and GOBLET portals.

## Results and Discussion

### Material sharing and reusability

Developing a strategy for sharing training materials is a key step towards reusability. The first step of this approach is the identification of training materials that should be shared and in which modality.

Materials that most trainers want to share include, but are not limited to, presentations, hands-on practicals, and datasets. Presentations are rarely reused as they are. Typically, trainers would use this type of material as a source of inspiration, to see how other instructors cover a particular topic, ending up reusing a subset of slides or just following the overall structure to then create their own set of slides. Tutorials instead tend to be reused in their original format; therefore, their completeness and consistency are crucial. Obtaining a well-documented tutorial would substantially speed up course preparation and trainers were unanimous in indicating that finding adequate datasets (in terms of size, content, and relevance to the audience) is often challenging. Datasets should be derived from real experiments, as opposed to simulated data; they need to be publicly available and also suitable to demonstrate particular analysis steps and their caveats. Moreover, they should be reasonable in size to allow for the fast execution of a typical HTS pipeline and be well annotated.

The ideal solution for sharing materials among trainers would be to build a unified collection of consistently annotated materials, easy to search and expand, and link this collection to existing training portals for the benefit of the trainers community at large. Such a collection would provide a framework (i) where trainers could share ideas, (ii) where materials could be tested, improved, or further developed in a collaborative manner, and (iii) where iterative versions of the same material could be archived.

Solutions to achieve these goals, and to address most of the issues discussed in the introduction, have already been developed in other contexts, e.g., software carpentry (http://software-carpentry.org/lessons/). It is common practice in computer science to collaboratively document, develop, test, and version programs. Based on this observation, it was decided to draft and develop a common collection of materials using a concurrent versioning system, namely Git [10], as the backbone.

To address the reusability issue, a core set of descriptors and a controlled vocabulary were devised and implemented as part of the chosen annotation strategy.

### Standards for describing training materials

Utilizing a minimal set of descriptors will allow for training materials that have been developed independently to be annotated in a consistent manner. The aim of such metadata would be to summarize basic information about the training material, describing its content, suitability for different audiences, and provenance, capturing all the information that we consider essential for the materials to be reused by someone else rather than the original author.

In particular, clear and concise learning objectives (LOs) should be adopted to describe courses and annotate training materials. LOs should help trainers to identify materials that they might want to reuse, to plan a course based on what individuals need to be able to achieve by the end of it, and also to track learning progression throughout a course. LOs should also help trainees to gather if a course or training materials are suitable for their needs and what they can expect to learn by attending a course or utilizing training materials.

The minimal set of descriptors agreed upon to describe each training material is described in Table 2 (including exemplary usage of the descriptors and, for the dataset descriptor, the exemplary use of a study by Buecker et al. [11]).

**Table 2:** The devised minimal set of descriptors for training materials.

Descriptor	Content	Example
Title		ChIP-Seq tutorial
Contact details of the author	Name and email address	
Content description/aims	A brief description of the content covered in the training material and overall aims	In this course we provide a basic introduction to conducting ChIP-Seq data analysis using the Galaxy framework. We will be retracing most of the steps required to get from an Illumina FASTQ sequence file all the way to performing peak calling and identifying over-represented sequence motifs and functional annotation. The aim is to give biologists the tools to independently run a basic analysis of ChIP-Seq data.
Target audience		Wet lab biologists with no or little programming experience
Learning objectives	Provide the trainees with an indication of what they should know/be able to do upon completion of the selected training module;	Develop an appropriate experimental design. Describe and perform the steps of a basic ChIP-Seq analysis workflow. Visualize data and results at various stages of the analysis. Annotate results. Critically assess data and results.
Prerequisites	Any prior knowledge that might help the participants to achieve the LOs	Working knowledge of Galaxy
Content	List of all files associated with the training material. For each file, the approximate length of time required to deliver the training and the IT/software requirements for running the tutorials should be indicated	Presentations, tutorials
Datasets	Should include: a description of the dataset, its provenance (including links to where the dataset can be found), how was it modified from its original form (if applicable, also include the code used to modify the dataset), and what can be demonstrated by using this particular dataset	In this practical we aim to identify potential transcription factor binding sites of Oct4 in mouse embryonic stem cells [11]. The original dataset can be downloaded from: http://www.ebi.ac.uk/arrayexpress/experiments/E-GEOD-56138/. Data were aligned to the mouse genome (mm10) and only those aligning to chromosome 1 were used for the practical.
Stability	Stability of the module’s content, e.g., how many times has this material been used in the classroom and when it was last updated	
Literature references		

Once the minimal set of descriptors was agreed upon, the training workflows for which training materials were available at the workshop were selected and, for each workflow’s module, materials were collaboratively annotated.

### Training workflows

Three workflows were selected, “RNA-Seq,” “ChIP-Seq,” and “variant analysis,” alongside two general topics: “Prerequisites” and “NGS-Introduction,” which provide the basic building blocks for the three workflows.

“Prerequisites” includes materials covering basic skills and knowledge in programming and statistics. For example, familiarity with the Unix shell and the R environment are crucial prerequisites. Depending on the target audience, introductions to Unix and R should be incorporated into a course’s program or training materials, possibly utilizing materials developed by the Software Carpentry Foundation [12,13]. “NGS-introduction” covers common concepts such as sequencing platforms, sequencing applications, and data formats.

The three workflows, “RNA-Seq,” “ChIP-Seq,” and “Variant calling,” aim to provide introductory as well as advanced training for these types of HTS analysis.

Each workflow was discussed and a set of modules was defined for each. As an example, the RNA-Seq topic contains the following modules: Pre-processing, Alignment, Alignment Quality Control (QC), Feature summarization, Feature summarization QC, Exploratory analysis, De-novo transcriptome assembly, and Differential Expression (DE). All the topics, their modules, and corresponding description are detailed in Table 3. The modules were furthermore grouped into essential and optional, highlighting those that are fundamental for each analysis workflow over others that can be used at the discretion of the trainer.

**Table 3:** Teaching topic core modules, as established by the NGS Trainers Consortium during the “Best Practice in HTS data analysis” workshop.

Topic	Level	Module Name	Module objectives	Essential /Optional
Prerequisite	Basic	Linux	Develop a familiarity with the Linux command line environment, such as navigating folders, learn what commands and parameters are and how to use them, open files on a terminal.	Essential
		R-programming	Describe the R programming environment. Perform basic programming in R	Essential
		Statistics	Review the basic statistical concepts	Essential
HTS Introduction	Basic	Technologies	Define the principles of high throughput sequencing technologies	Essential
		Data Formats	Describe the different data formats commonly used for HTS data	Essential
ChIP-Seq	Advanced	Preprocessing	Describe necessary preprocessing steps, Perform a Quality Assessment and interpret the results	Essential
		Alignment	Perform alignment, Discuss alignment considerations	Essential
		ChIP-Seq-QC	Explain ChIP-Seq specific QC steps, Perform ChIP-Seq QC on a data set	Essential
		Peak-calling	List appropriate peak-calling software, Describe the theoretical basis of peak-calling, Apply different peak callers	Essential
		Visualization	Visualize raw and processed data, Assess data quality	Essential
		Annotation	Interpret results in the genomic context	Optional
		Differential binding (DB)	Perform DB analysis, Interpret the output, Recognize the need for normalization	Optional
		Working with biological replicates	Compare and combine different biological replicates using IDR analysis	Optional
		Non-peak based analysis	Inspect signal around regions of interest, Generate carpet plots	Optional
RNA-Seq	Advanced	Preprocessing	Apply QC software and interpret the output, Decide/perform necessary preprocessing steps	Essential
		Alignment	Distinguish between genome and transcriptome alignment, Select the appropriate tool, Recognize the challenges and pitfalls, Produce alignment, Interpret the alignment file	Essential
		Alignment QC	Apply QC software, Interpret the output, Decide/perform necessary filtering steps	Essential
		Feature summarization	Produce a table of counts, Identify a proper strategy for the biological question, Interpret output	Essential
		Exploratory analysis	Visualize alignments, Applying QC software (e.g. clustering, Principal Component Analysis (PCA), etc.), Identify confounding effects and take necessary action	Essential
		De-novo transcriptome assembly	Perform the analysis, Recognize the challenges and pitfalls, Interpret the assemblers output	Optional
		Differential expression (DE)	Perform DE analysis, Interpret the output, Recognize the need for normalization and dispersion estimation	Optional
Variant analysis	Advanced	Preprocessing	Apply QC software and interpret the output, Decide/perform necessary preprocessing steps	Essential
		Alignment	Differentiate genome and transcriptome alignment, Select the appropriate tool, Recognize the challenges and pitfalls, Produce alignment, Interpret the aligners output	Essential
		Alignment QC	Apply QC software and interpret the output, Decide/perform necessary filtering steps	Essential
		Variant Calling	Apply variant calling software, Understand the format and the different type of variants	Essential
		Variant Analysis	Visualize variant calls, Interpret the output, Select/filter variants	Essential
		Annotating Variants	Annotate variants, Lookup potential effects on coding regions, Evaluate putative clinical relevance	Optional

The level column details what the target audience of individual topics should be. The optional modules are the ones that can vary based on the course program and duration. The essential modules are the ones that are considered mandatory for any course addressing the corresponding topic; i.e., they are the core set of modules, common to all downstream analyses, that a trainee must learn about.

A set of controlled vocabularies—adapted from the EMBRACE Data and Methods (EDAM) ontology [14]–was selected to tag individual modules in order to facilitate annotation and enable keyword-based searching of the repository content.

All materials provided by the trainers attending the workshop were assigned to the appropriate workflow module, annotated as described in the previous session, and uploaded to a Git repository.

### Repository implementation

**Fig 1:**
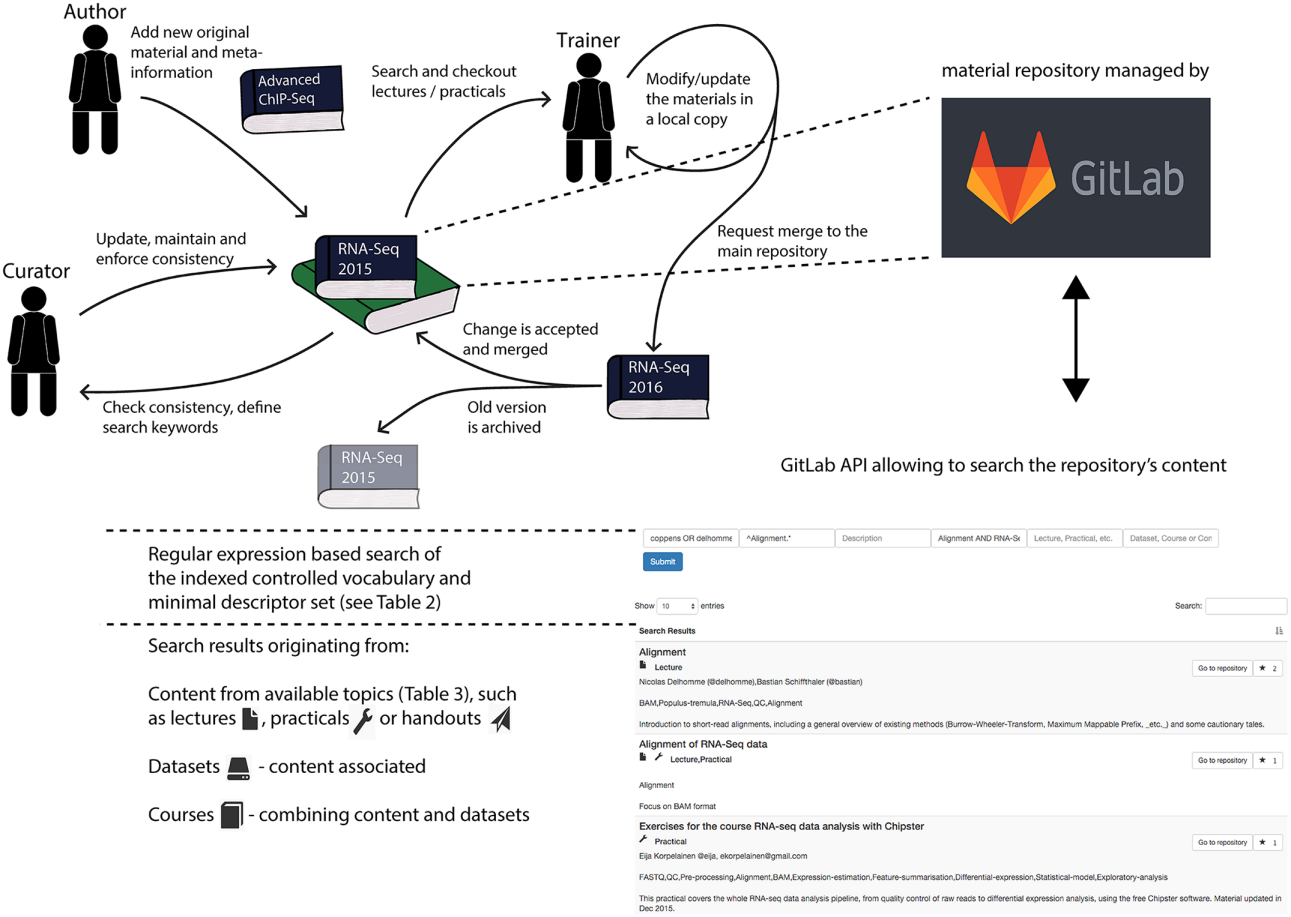
Overview of the repository’s implementation, content, search interface, and flowchart of roles and actions. Authors add their material to the main repository, where they can be searched and retrieved by trainers. Any trainer can then build on existing materials for their own lectures and practicals and request a merge of the updated materials into the main repository, which upon validation, results in the older version of the material to be archived. Two of the consortium members act as curators for each topic, ensuring the completeness of the descriptors and the adequate use of the controlled vocabularies. The newly added materials are then indexed and made discoverable through the search interface. Both the search interface and repository rely on a GitLab instance hosted by the consortium. The Git logo is licensed under CC BY-SA 4.0 by GitLab (https://about.gitlab.com/press/).

The current implementation of the HTS training materials repository has two components: a back-end, based on Git to handle the content, and a front end [15], served as web-content, which builds on the Git repository, as detailed in Fig 1.

The Git versioning system is used to keep track of materials’ updates, in the same way as it is used in software development to keep track of changes applied to source code.

Contributors who wish to apply changes to the repository content can do so by forking it. Modified materials can then be reloaded, after passing consistency checks in order to prevent accidental data modification or deletion. Additionally, the API associated with the selected Git implementation (GitLab [16]) allows for the development of applications that programmatically access the materials and their metadata. This API was used to: (i) index the module metadata repository content and (ii) program the search functionality, which is available from the repository’s landing page. Leveraging on such metadata, a trainer can query the repository based on their teaching interests and retrieve relevant training materials. Finally, to ensure an easier navigation within the repository, hyperlinks are used to connect related materials, e.g., to collate all the material used in a given course.

### Contributing to the repository

The project’s wiki provides contributors with templates for the submission of new materials, the set of descriptors (i.e., the controlled vocabulary necessary to tag materials), and submission instructions, including an introduction to the minimal set of Git commands essential to contribute to the repository. Trainers who wish to contribute to the repository can do so either programmatically or by interacting with a curator. In either case, they need to provide the materials and the corresponding metadata (see descriptors in Table 2). For a programmatic submission, the contributor can either login via an existing Google or GitHub account or create a new repository’s user account and then follow the afore-mentioned instructions. If necessary, e.g., for a novel type of data or analysis, descriptors could be extended. For interactive submissions, we provide online forms, available from the repository’s landing page, to create the material’s metadata. Upon successful generation of the metadata, the contributor will be contacted by a curator for completing the submission. Regardless of the submission approach, new materials are checked for annotation inconsistencies before being made publicly available.

During the initial testing phase, the repository was met with great interest from the community. Currently, 47 members have created an account in the system, of which 29 have attended the workshop and 28 have submitted materials. To facilitate communication between contributors, we have set up a mailing list, which currently includes 26 people.

## Conclusions

We have developed a strategy for the curation of HTS training materials and established a working framework for the sharing of such materials among trainers for promoting and strengthening interactions among them and learning from each other's teaching experience.

The Git repository of curated HTS materials that we have created is now publicly available at http://bioinformatics.upsc.se/htmr and discoverable through both ELIXIR and GOBLET training portals. It now provides the potential to ease the preparation of training courses via a community driven sharing strategy. In addition, it enables trainers to update and modify their material while keeping track of the changes. This solution is scalable and has been made robust through the use of an easily manageable API in combination with consistent curation.

Community initiatives are already planned to refine the training materials curation strategy and extend the coverage of this collection.

ELIXIR has recently organized a thematic hackathon focusing on the use of the EDAM ontology to annotate training materials currently available in the collection. The workshop had the dual purpose of tagging materials with EDAM ontology annotations and, at the same time, improving the ontology’s coverage. Additionally, training materials were mapped to the bioinformatics tools and resources from the ELIXIR’s Tool and Data Service registry [17,18] to increase the simultaneous discoverability of bioinformatics tools and related training materials.

GOBLET and ELIXIR are now planning a second workshop that will bring together trainers working in the field of metagenomics, to enhance the training network within this field. The strategy presented in this paper will be applied to the curation of existing metagenomics analysis training materials, with the aim to define a generic approach for the curation and dissemination of training materials through training portals such as TeSS and GOBLET.

We encourage trainers active in delivering HTS training, as well as trainers that might be new to this field, to get involved, utilize the materials already available in the repository to deliver training in this area, and contribute to this initiative with new materials.

## Supporting Information

S1 Table NGS Trainers Consortium members and affiliations. (XLSX)
